# Adult Presentation of Congenital Mitral Stenosis: The Challenges of a True Parachute Mitral Valve

**DOI:** 10.1055/a-2785-8203

**Published:** 2026-01-22

**Authors:** Maria Jose Lizano, Álvaro Herrera, Eduardo Induni, Félix Eduardo Solís, Tulio Caldonazo

**Affiliations:** 1 Department of Cardiothoracic Surgery, Costa Rican Social Security, Hospital México, San José, Costa Rica; 2 Department of Cardiology, Costa Rican Social Security, Hospital México, San José, Costa Rica; 3 Department of Cardiothoracic Surgery, Friedrich-Schiller-University Jena, University Hospital, Jena, Germany

**Keywords:** congenital mitral stenosis, parachute mitral valve, mitral valve replacement

## Abstract

**Background:**

Parachute mitral valve (PMV) is a rare congenital anomaly where all chordae tendineae insert into a single papillary muscle, causing stenosis or regurgitation. Adult presentations are uncommon and often underdiagnosed.

**Case Description:**

A 35-year-old male presented with exertional dyspnea. Echocardiography demonstrated severe mitral stenosis, reduced ejection fraction, a bicuspid aortic valve, and pulmonary hypertension. Intraoperative findings confirmed true PMV. Due to anatomical complexity, a 27-mm mechanical prosthesis replacement was performed successfully, with rapid postoperative recovery.

**Conclusion:**

Adult PMV requires high clinical suspicion and often surgical confirmation. Valve replacement is effective in complex cases, emphasizing the importance of early diagnosis and intervention.

## Introduction

The mitral valve is composed of two leaflets: A larger anterior leaflet that extends vertically and a smaller posterior leaflet that expands transversely, separated by two commissures. For proper closure, alignment between the surface area of the leaflets and the orifice area is crucial. The anterior leaflet connects to the left ventricular outflow tract, while the posterior leaflet is anchored to the muscular base of the left ventricle. The papillary muscles, classified as posteromedial and anterolateral, attach to the ventricular wall. A rare condition known as “parachute mitral valve” (PMV) can occur in adulthood, with only a few documented cases in the past 50 years. In this condition, all chordae tendineae attach to a single papillary muscle, which may result from the fusion of two muscles or involve only the typically hypoplastic posterior muscle. This parachute valve often presents with stenosis due to interchordal space obliteration from excess valvular tissue and may also lead to regurgitation due to a large functional orifice near the hypoplastic papillary muscle.

## Case Presentation

**Fig. 1:**
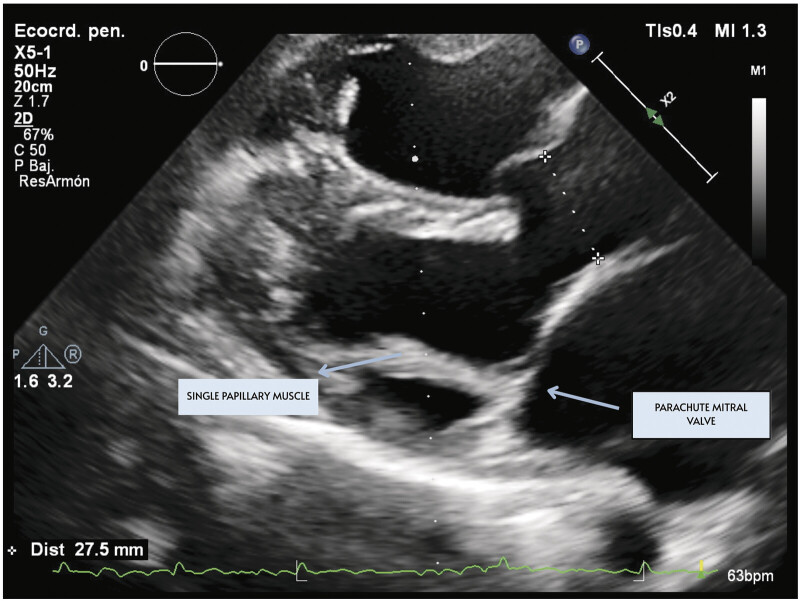
Echocardiographic view showing the mitral valve with chords attached to a single papillary muscle.

A 35-year-old Costa Rican male with a history of hypertension, dyslipidemia, hepatic steatosis, tinea versicolor, and atrial flutter presented with dyspnea while riding a horse, with no episodes of paroxysmal nocturnal dyspnea or orthopnea. A grade 3 holodiastolic murmur was noted during a clinic visit, and an echocardiogram revealed the following: Severe congenital mitral stenosis (peak: 27 mm Hg, mean: 15 mm Hg); dilated left atrium, inflow velocity in the left atrial appendage: 29 cm/s; left ventricle with normal dimensions but with diffuse hypokinesia ( Fig. 1 ). Reduced ejection fraction (Left Ventricular Ejection Fraction [LVEF] 30%), associated with an atrial septal aneurysm with a patent foramen ovale, and the presence of a ventricular septal defect of 17 mm with aneurysmal tissue obstructing flow through the defect, with no evidence of septal shunts noted. Additionally, as part of his congenital spectrum, he has a bicuspid aortic valve, with no signs of stenosis, insufficiency, or aortopathy at this time. Data indicate right heart failure, with a dilated right ventricle and fractional area change (FAC) 28%. The day before surgery, he was moved to the intensive care unit (ICU), where a Swan–Ganz catheter was placed, indicating a pulmonary capillary wedge pressure of 28 mm Hg, a central venous pressure of 9 mm Hg, and a pulmonary artery systolic pressure of 57 mm Hg.

**Fig. 2:**
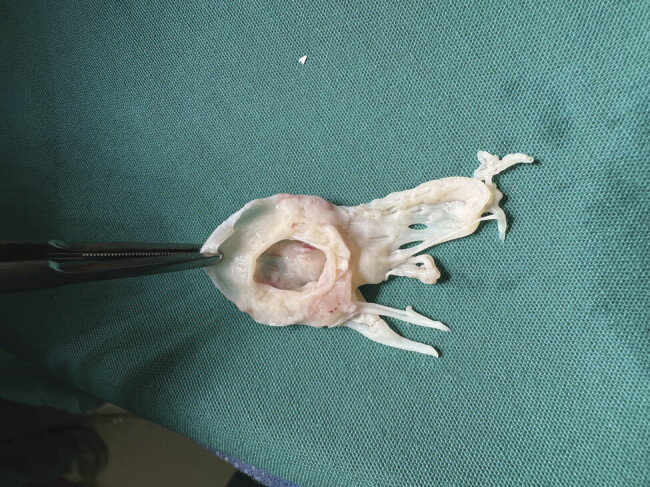
Mitral valve with true parachute morphology, intraoperative biological photo.

**Fig. 3:**
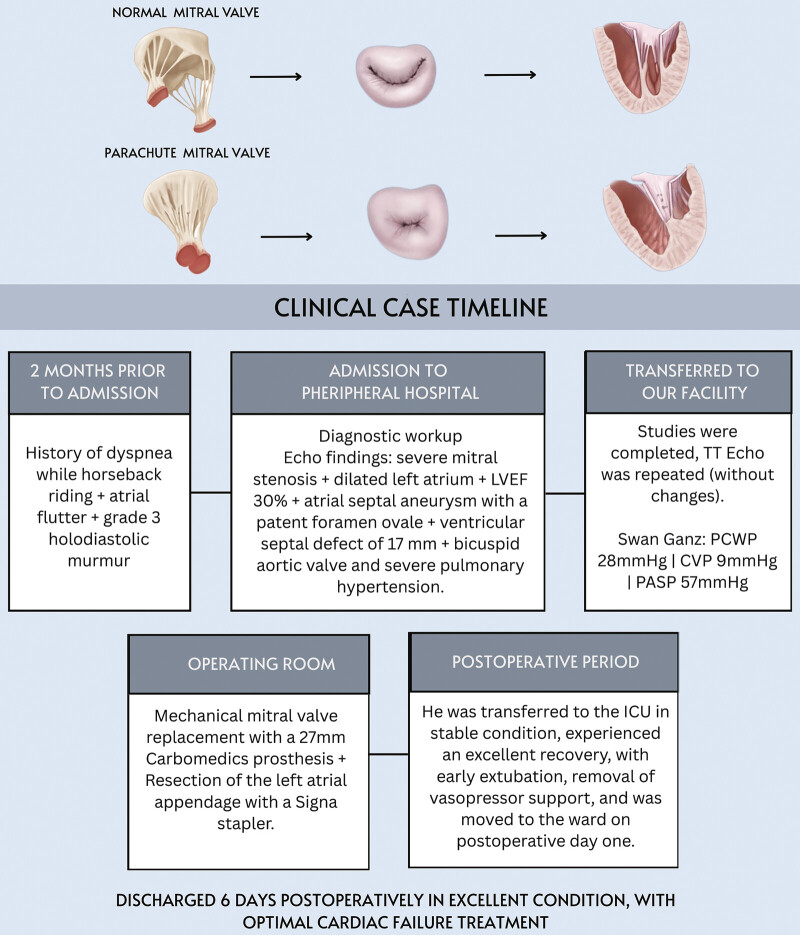
The timeline summarizes key events: Progressive dyspnea and atrial flutter, diagnostic confirmation of severe mitral stenosis with associated anomalies, surgical replacement with a 27-mm Carbomedics prosthesis plus left atrial appendage resection, and an uncomplicated recovery with discharge on postoperative day 6.

The surgery involved extracorporeal circulation with arterial cannulation in the ascending aorta and venous bicaval drainage, ensuring myocardial protection through both antegrade and retrograde cardioplegia. A left atriotomy was performed, exposing the stenotic mitral valve with a true parachute morphology ( Fig. 2 ). Sutures were placed in the native annulus, and a 27-mm Carbomedics prosthesis was secured. After tying the sutures, the atriotomy was closed, and intraoperative echocardiography confirmed functionality and absence of valvular leaks. The left atrial appendage was resected with a Signia stapler, and the patient was weaned off cardiopulmonary bypass after 17 minutes. Initially, the patient required pacemaker support, nitric oxide, norepinephrine, and dobutamine, which were gradually reduced during the immediate postoperative period. He was transferred to the ICU in stable condition, experienced an excellent recovery, with early extubation, removal of vasopressor support, and was moved to the ward on postoperative day 1. Discharged 6 days postoperatively in excellent condition, with optimal cardiac failure treatment ( Fig. 3 ).

## Discussion

The concept of PMV was first described in 1963 as a unique mitral valve anomaly where all chordae tendineae connect to a single papillary muscle, resulting in a funnel-shaped structure. This condition likely arises from abnormal segmentation of the papillary muscle precursor during the 5th to 19th weeks of gestation, leading to fusion into a single muscle. 1 In typical PMV cases, the chordae are shortened and thickened, with the posteromedial papillary muscle intact, while the anterolateral muscle is absent. 2 PMV can present as an isolated defect or as part of a more complex condition known as Shone syndrome, which includes additional congenital anomalies like supravalvular mitral membranes and subaortic stenosis. 3 4 5 6 About 25.9% of patients with severe congenital mitral stenosis have an associated PMV, while Shone syndrome occurs in approximately 1.17% of congenital heart defects. 2

Current evidence indicates that PMV is relatively infrequent in adult patients and may appear in isolation. In adulthood, cases of PMV typically involve milder conditions that often remain undetected until later in life. This may be due to the fact that complex congenital lesions usually become apparent early and necessitate multiple surgical corrections, carrying a high risk of mortality. Consequently, adults with PMV may represent a smaller population with milder manifestations of the condition, frequently remaining undiagnosed until adulthood. Additionally, asymptomatic adults may not undergo echocardiographic evaluations, further contributing to underdiagnosis. Even among those who receive echocardiography, the diagnosis of PMV is not always confirmed. 4

Although the embryologic origins of parachute-like mitral valves differ from those of true PMV, their clinical presentations often resemble one another, particularly in cases where the mitral valve apparatus shows significant asymmetry, leading to mitral stenosis and regurgitation. Parachute-like mitral valves have been linked to many similar concomitant defects as those associated with PMV, such as the bicuspid aortic valve. Additionally, they have been associated with abnormal pulmonary venous return, persistent left-sided superior vena cava, double-outlet right ventricle, fibroelastosis, and pulmonary atresia. 7 8

Echocardiography is the primary diagnostic modality for PMV, achieving effective identification in approximately 77.77% of cases. The mitral valve's characteristic deformity, known as the parachute configuration, is optimally visualized using parasternal short-axis views of the left ventricle, where a single papillary muscle is identifiable at mid-level. The distinctive “parachute leaflets” can also be seen in the basal short-axis view. Furthermore, a long-axis view of the left ventricle can confirm the presence of a single papillary muscle from which all chordae tendineae arise. 4

It is essential to recognize that PMV may be mistakenly identified as conditions, such as pseudo-parachute or parachute-like mitral valves, where the chordae attach to major papillary muscles, leaving one hypoplastic and adjacent to the primary muscle. Comprehensive evaluation through echocardiography, often necessitating transesophageal approaches, can assist in distinguishing true PMV from its mimickers. In some cases, the specific morphology may only be established during surgical intervention. 4

Regarding therapeutic options, PMVs are rarely isolated anomalies, leading many patients to require multiple surgical interventions and a high reintervention rate. In some instances, PMVs can function adequately without surgical intervention. However, if the hemodynamic gradient increases, the presence of a supravalvular ring should be considered. Most isolated PMVs are amenable to mitral valve repair, while replacement is reserved for those with severe mitral valve damage. Some stenotic PMVs have been repaired via an incision in the papillary muscle and leaflet fenestration. In children, MVR poses challenges, including high operative mortality rates, occurrences of complete heart block requiring pacemaker insertion, and the unavailability of appropriately sized prosthetic valves for growth, as well as complications related to postoperative anticoagulant management and rapid deterioration of bioprosthetic valves. 2 6

In our case, given the patient's reduced ejection fraction, significant ventricular dilation, and severe pulmonary hypertension, we determined that mitral valve replacement was the best option for favorable outcomes to reduce circulatory arrest and cross-clamp times to ensure a successful extracorporeal circulation pump exit.

## Conclusion

PMV is a rare congenital cardiac anomaly that can lead to mitral stenosis and pulmonary hypertension. The present case describes an adult male who presented with heart failure and low ejection fraction with severe pulmonary hypertension, and was found to have congenital mitral stenosis in the context of an isolated PMV. Echocardiography played an important role in the diagnostic workup; although the diagnosis was made intraoperatively, surgical resection was necessary, and a mechanical prosthesis was successfully placed without complications.
